# A neuroimmune hypothesis linking chronic cervical inflammation to systemic symptoms: the role of the vagus–NTS axis

**DOI:** 10.3389/fneur.2026.1881152

**Published:** 2026-10-08

**Authors:** Min-Chieh Chang

**Affiliations:** Chang Min-Chieh Clinic, Kaohsiung, Taiwan

**Keywords:** burning mouth syndrome, central sensitization, cervical inflammation, chronic cervicitis, dyspareunia, glossodynia, neuroimmune axis, nucleus tractus solitarius

## Abstract

Chronic pain syndromes in women often present with symptoms that do not fully correspond to the anatomical site of detectable pathology. Dyspareunia, vulvar pain, bladder discomfort, glossodynia, headache, dizziness, gastrointestinal symptoms, palpitations, insomnia, and musculoskeletal pain may coexist, suggesting shared neuroimmune and autonomic mechanisms rather than isolated local disease alone. This hypothesis was motivated by two preliminary clinical observations presented at meetings of the Taiwan Association of Obstetrics and Gynecology. The first series, presented in March 2024, involved 28 women with dyspareunia in whom the anatomical location of pain did not consistently correspond to the presumed pathological origin, with symptomatic improvement observed after targeted endocervical treatment. The second series, presented in March 2026, involved 28 women with intractable glossodynia, several of whom had coexisting vulvovaginal or cervical inflammatory findings and reported improvement after gynecologic treatment. These observations are not presented as definitive evidence of causality, but as hypothesis-generating clinical signals. We propose that chronic cervical inflammation may act as a hidden peripheral inflammatory source capable of modulating vagal afferent signaling to the nucleus tractus solitarius. Through interactions with autonomic, hypothalamic, limbic, and cortical networks, persistent cervical inflammatory input may contribute to neuroimmune amplification and systemic symptoms beyond the anatomical location of pain. This model is testable through prospective studies evaluating cervical inflammatory markers, heart rate variability, vagal tone, cytokine profiles, central sensitization indices, and symptom changes after targeted cervical treatment. Pain location may not necessarily indicate pathological origin, and chronic cervical inflammation may represent an underrecognized contributor to systemic neuroimmune symptoms through the vagus–NTS axis.

## 1 Introduction

Pain is commonly interpreted according to its anatomical location. In clinical practice, tongue pain is usually investigated as an oral or dental disorder, vulvar pain as a local vulvar disease, bladder discomfort as a urological disorder, and dyspareunia as a pelvic or sexual pain condition. Although this anatomical approach is clinically useful, it may be insufficient in chronic, recurrent, or medically unexplained pain syndromes, in which symptom location may not fully reflect the underlying pathological origin.

Chronic pain syndromes frequently involve complex interactions among peripheral inflammation, sensory nerve activation, central sensitization, autonomic dysregulation, and altered interoceptive processing (32). In women, chronic pelvic pain, dyspareunia, vestibulodynia, bladder pain syndrome, and vulvar pain often coexist with extra-pelvic symptoms such as headache, gastrointestinal discomfort, sleep disturbance, fatigue, musculoskeletal pain, and oral burning pain. Such multisite symptom clustering suggests that chronic pain may not always arise from isolated local tissue pathology, but may instead reflect shared neuroimmune and autonomic mechanisms.

The vagus nerve is a major bidirectional communication pathway between the viscera and the brain. Vagal afferents transmit interoceptive and inflammatory information to the brainstem, particularly to the nucleus tractus solitarius (NTS), which integrates visceral, immune, autonomic, and behavioral responses (1–3). Experimental neuroanatomical studies in rodents have demonstrated vagal afferent connections involving the uterus and cervix and projections to the brainstem (4). Electrophysiological and tracing studies have additionally demonstrated bidirectional vagus–uterus communication (5). These findings provide a biological basis for considering a possible cervix–vagus–brainstem communication pathway.

Based on preliminary clinical observations and existing neuroimmune concepts, we propose a hypothesis in which chronic cervical inflammation may act as a hidden peripheral inflammatory source capable of influencing systemic symptoms through vagal afferent signaling and NTS-mediated central autonomic integration. This manuscript is intended as a hypothesis-generating clinical and mechanistic framework, not as a definitive causal report.

## 2 Clinical observations leading to the hypothesis

The present hypothesis was clinically motivated by two preliminary case-series observations presented at meetings of the Taiwan Association of Obstetrics and Gynecology (TAOG). These observations are described here as hypothesis-generating signals rather than definitive evidence of causality.

### 2.1 TAOG March 2024: dyspareunia and discordance between pain location and presumed pathological origin

The first clinical observation, presented in March 2024, involved 28 women with dyspareunia. These patients presented with sexual pain in various anatomical locations, including superficial vulvar or vestibular pain, deep pelvic pain, vaginal pain, or mixed pain patterns. However, the anatomical location of pain did not consistently correspond to the presumed pathological origin.

In this preliminary series, targeted endocervical treatment was associated with symptomatic improvement in many patients. This observation raised the possibility that chronic endocervical inflammation may contribute to pelvic pain amplification beyond the apparent site of pain. The central clinical message derived from this series was that pain location may not necessarily indicate pathological origin.

In addition to dyspareunia and pelvic pain, a subset of patients reported symptoms extending beyond the pelvis, including headache, dizziness, gastrointestinal complaints, sleep disturbance, urinary symptoms, and musculoskeletal discomfort. These coexisting manifestations were not the primary endpoints of the original conference series and were not systematically quantified in all participants; therefore, they should be interpreted as exploratory clinical observations rather than established treatment outcomes. Nevertheless, their coexistence with chronic pelvic symptoms contributed to the clinical impression that a focal pelvic inflammatory condition might be associated with a broader symptom phenotype.

### 2.2 TAOG March 2026: intractable glossodynia and coexisting lower genital tract inflammation

The second clinical observation, presented in March 2026, involved 28 women with intractable glossodynia. Glossodynia, or burning mouth syndrome, is anatomically distant from the lower genital tract. However, several patients in this series were observed to have coexisting vulvovaginal or cervical inflammatory findings. Some patients reported improvement in tongue pain after gynecologic treatment directed at the lower genital tract.

This observation was clinically important because the symptomatic site—the tongue—was anatomically distant from the presumed peripheral inflammatory source. The apparent dissociation between symptom location and the site of treatment extended the concept generated by the dyspareunia series beyond the pelvis. It raised the possibility that persistent peripheral inflammatory input might influence pain processing through mechanisms involving neuroimmune signaling, autonomic regulation, and central sensitization rather than through direct anatomical continuity alone.

Glossodynia is increasingly discussed as a neuropathic, nociplastic, or centrally mediated pain condition. Therefore, the coexistence of glossodynia with lower genital tract inflammatory findings does not establish that cervical inflammation caused the oral pain. Instead, this clinical pattern provided a second, anatomically distant observation that contributed to the development of the present neuroimmune hypothesis.

The original clinical observations were not designed to systematically quantify all neurological, gastrointestinal, urinary, autonomic, sleep-related, or musculoskeletal symptoms. Consequently, the present manuscript does not interpret these case series as proof of a defined multisystem syndrome. Rather, they are presented as hypothesis-generating observations that motivated subsequent consideration of a broader systemic symptom phenotype.

### 2.3 Interpretation of the two observations

Together, these two preliminary clinical observations suggest that chronic cervical or lower genital tract inflammation may act as a hidden peripheral inflammatory source capable of modulating neuroimmune signaling beyond the local pelvic region. The 2024 dyspareunia series supports the concept that the anatomical site of perceived pain may differ from the site of the underlying pathology. The 2026 glossodynia series extends this concept beyond the pelvis, suggesting that cervical or lower genital tract inflammation may participate in distant or systemic symptom manifestations. These key clinical observations are summarized in Table 1.

**Table 1:** Clinical observations leading to the hypothesis.

Observation	Clinical condition	Key finding	Hypothesis–generating implication
TAOG March 2024	28 women with dyspareunia	Pain site did not consistently match presumed pathological origin; improvement after targeted endocervical treatment	Chronic cervical inflammation may contribute to pelvic pain amplification beyond the apparent pain location
TAOG March 2026	28 women with intractable glossodynia	Coexisting vulvovaginal or cervical inflammatory findings; some improvement after gynecologic treatment	Lower genital tract inflammation may be linked to extra-pelvic pain through neuroimmune pathways

An additional clinically relevant observation is that treatment response was not uniform among patients. Although patients in the original clinical series generally showed some degree of improvement, the magnitude, timing, and anatomical distribution of symptom improvement varied. Some symptoms improved more markedly or earlier than others, and the pattern of improvement differed between individuals.

The original clinical series were not prospectively designed to systematically quantify autonomic symptoms or to compare predefined patterns of treatment response using mechanistic measures. Therefore, these observations do not allow firm conclusions regarding the factors responsible for individual differences in treatment response.

An important clinical feature was that treatment response was not simply dichotomous. Patients generally showed some degree of improvement, but the timing, magnitude, and anatomical distribution of symptom improvement varied. In some patients, the chief complaint improved gradually, whereas associated symptoms such as dizziness, tinnitus, gastroesophageal reflux, or musculoskeletal pain improved earlier or more prominently. Thus, a patient could demonstrate a meaningful clinical response in one symptom domain even while the primary complaint remained only partially improved. This pattern suggests that treatment response may involve multiple symptom networks rather than a single uniform endpoint.

We hypothesize that differences in baseline central sensitization, autonomic regulation, and neuroimmune responsiveness may influence the degree and pattern of clinical improvement through the proposed neuroimmune pathway. These possibilities should be examined prospectively using predefined clinical, autonomic, and sensitization measures.

These observations do not establish causality but provide a clinical rationale for developing and testing the proposed neuroimmune hypothesis.

## 3 Hypothesis

We propose that chronic cervical inflammation may serve as a hidden peripheral inflammatory source capable of activating or modulating afferent neuroimmune signaling. This input may be transmitted through pelvic afferent pathways and vagal afferent pathways to the nucleus tractus solitarius and related central autonomic networks. Through downstream interactions with the hypothalamus, limbic system, cortical interoceptive regions, and descending pain modulatory systems, persistent cervical inflammatory input may contribute to systemic symptoms beyond the anatomical location of pain.

The proposed pathway can be summarized as follows:

**Chronic cervical inflammation** **→** **local cytokine and immune activation** **→** **pelvic and vagal afferent signaling** **→** **nucleus tractus solitarius** **→** **autonomic, hypothalamic, limbic, and cortical modulation** **→** **systemic neuroimmune symptoms**

This hypothesis does not imply that all systemic symptoms in women originate from the cervix. Rather, it suggests that in a subset of patients, chronic cervical inflammation may function as an overlooked contributor to neuroimmune amplification, central sensitization, and autonomic dysregulation.

## 4 Biological plausibility

### 4.1 Cervical inflammation as a persistent peripheral immune signal

Chronic inflammation in mucosal tissues can generate persistent immune signaling through cytokines, chemokines, prostaglandins, immune-cell activation, and local nociceptor sensitization (6, 7).

In the cervix, chronic endocervical inflammation may remain clinically underrecognized because symptoms can be nonspecific, intermittent, or anatomically misleading. Instead of causing only local discharge, contact bleeding, or pelvic discomfort, chronic cervical inflammation may provide low-grade but persistent afferent input to pelvic and autonomic circuits.

From a neuroimmune perspective, persistent peripheral inflammatory input can lower sensory activation thresholds and produce peripheral sensitization. When such afferent input persists, spinal and supraspinal nociceptive networks may become increasingly responsive, contributing to central sensitization and symptom amplification (7–9).

### 4.2 Vagal afferents from the uterus and cervix

The vagus nerve is classically known for its role in cardiovascular, respiratory, gastrointestinal, and immune regulation. Neuroanatomical evidence in rodents has demonstrated vagal afferent connections involving the uterus and cervix and projections toward the brainstem (4). Ortega-Villalobos et al. (5), using electrophysiological recordings and horseradish peroxidase tracing, further demonstrated a bidirectional vagal complex–uterus connection in rats. In addition, Hubscher and Berkley (10) showed that neurons in the rat NTS respond to stimulation of the uterus, cervix, and vagina, with vagotomy modifying these responses, supporting participation of vagal input in reproductive-tract sensory processing. These experimental findings provide an anatomical and functional foundation for a possible uterus/cervix–vagus–brainstem pathway. However, these findings are derived primarily from animal studies, and the functional significance and relative contribution of cervicovagal signaling in humans remain uncertain. This finding is important because it provides an anatomical foundation for the possibility that cervical or uterine signals may reach central autonomic integration centers.

Although human clinical evidence remains limited, this animal neuroanatomical evidence supports the plausibility of a cervix–vagus–brainstem pathway. The present hypothesis builds on this anatomical foundation and extends it into a clinical framework linking cervical inflammation, vagal afferent signaling, NTS integration, and systemic symptom generation.

### 4.3 Nucleus tractus solitarius as a central integration hub

The nucleus tractus solitarius is a major brainstem relay center for visceral afferent input. It receives vagal sensory information and participates in autonomic, cardiovascular, respiratory, gastrointestinal, immune, and interoceptive regulation (1, 11, 12).

If chronic cervical inflammation provides persistent afferent input to the NTS, the NTS may act as an integration hub linking peripheral inflammation to systemic symptom expression.

Through its connections with the dorsal motor nucleus of the vagus, parabrachial nucleus, hypothalamus, amygdala, insula, and cortical networks, the NTS may influence autonomic tone, pain perception, arousal, sleep, gastrointestinal function, emotional regulation, and body-state awareness (1, 11, 12).

### 4.4 Vagus nerve and the inflammatory reflex

The vagus nerve is also a key component of the inflammatory reflex, a neural mechanism through which the nervous system can regulate immune responses (2, 3, 26). Vagal signaling can influence cytokine production and systemic inflammation through autonomic and cholinergic anti-inflammatory mechanisms. This bidirectional communication suggests that peripheral inflammation may be sensed by the nervous system and that neural pathways may, in turn, regulate immune activity.

In the context of chronic cervical inflammation, persistent immune activation may not remain confined to the local reproductive tract. Instead, it may be represented within central autonomic networks through afferent signaling, thereby contributing to systemic symptom perception and neuroimmune amplification.

### 4.5 Central sensitization and systemic symptom amplification

Central sensitization refers to increased responsiveness of nociceptive neurons and networks within the central nervous system to peripheral input (7, 8, 37). Repeated or persistent nociceptive and inflammatory input can produce activity-dependent changes in spinal and supraspinal pathways, including lowered activation thresholds, increased synaptic responsiveness, reduced inhibitory control, and enhanced responsiveness to subsequent sensory input. Clinically, these changes may contribute to hyperalgesia, allodynia, multisite pain, and a reduced correspondence between the anatomical location of symptoms and the initiating peripheral pathology (9).

Neuroimmune mechanisms may further sustain this sensitized state. Persistent peripheral inflammation can activate immune cells and promote the release of cytokines and other inflammatory mediators, which may enhance the excitability of peripheral sensory neurons and influence central nociceptive processing (6, 7). With continued afferent input, amplification may extend beyond the original spinal segments through interconnected brainstem, thalamic, limbic, and cortical networks involved in pain, interoception, arousal, and autonomic regulation.

Central sensitization has been described in chronic pelvic pain and urogynecological pain syndromes (13, 14), while burning mouth syndrome has also been investigated as a neuropathic or centrally mediated pain condition (15, 16). These observations support the broader concept that persistent peripheral input may contribute to symptom amplification at anatomically distant sites.

Within the framework proposed here, repeated inflammatory and afferent signaling arising from chronic cervical inflammation could, in susceptible individuals, contribute to such a sensitized central state. Pelvic afferent pathways may participate in spinal and cross-organ sensitization, whereas vagal afferent signaling and NTS-centered autonomic networks may provide an additional route through which persistent visceral and inflammatory information influences central processing. These pathways are not proposed to operate independently; rather, they may interact with neuroimmune signaling and supraspinal pain networks to produce a broader amplification state.

Such amplification could help explain why some patients experience combinations of pelvic pain, oral burning pain, urinary symptoms, gastrointestinal complaints, sleep disturbance, dizziness, palpitations, headache, and musculoskeletal pain. Importantly, this model does not imply that cervical inflammation directly causes each of these symptoms. Instead, chronic cervical inflammation is proposed as one possible persistent peripheral contributor capable of interacting with preexisting susceptibility to central sensitization, autonomic dysregulation, and neuroimmune amplification. This cervix-specific contribution remains hypothetical and requires prospective mechanistic validation.

## 5 Proposed clinical symptom spectrum

The proposed cervix–vagus–NTS hypothesis may help explain why, in a susceptible subset of women, symptoms can occur across pelvic, urinary, extra-pelvic pain, gastrointestinal, and autonomic domains. Importantly, the proposed mechanisms are not identical for all symptom groups. Rather, the model involves interacting pelvic afferent, cross-organ, neuroimmune, autonomic, and central sensitization pathways.

### 5.1 Pelvic pain symptoms

Dyspareunia, deep pelvic pain, vaginal pain, vestibulodynia, post-coital discomfort, and pelvic pressure may arise through persistent activation and sensitization of pelvic sensory pathways (28, 36, 37, 39). Chronic inflammatory input from the cervix may lower nociceptor thresholds and contribute to peripheral sensitization. Convergence of visceral and somatic afferent inputs within spinal pathways may further facilitate referred pain and pelvic pain amplification (17, 18, 27).

In addition to neural sensitization, local paracrine and neuroimmune signaling may contribute to pelvic symptom amplification. Inflammatory mediators released within the pelvic environment may influence nearby sensory nerve endings, immune cells, and adjacent pelvic tissues. Such local signaling, together with convergence of pelvic afferent pathways, could lower nociceptive thresholds and reinforce peripheral and cross-organ sensitization (19).

### 5.2 Urological symptoms

Bladder discomfort, urinary frequency, post-void suprapubic pain, urethral discomfort, and interstitial cystitis-like symptoms may be related, in part, to pelvic organ cross-sensitization. Convergent sensory processing among pelvic organs can allow persistent nociceptive input from one organ to modify the excitability of afferent pathways serving another organ (19–21). Within the present hypothesis, chronic cervical inflammatory input could therefore contribute to amplification of bladder-related symptoms without implying primary bladder pathology in every patient.

Local paracrine and neuroimmune signaling may provide an additional mechanism linking pelvic inflammation with urinary symptoms. Inflammatory mediators released from an affected pelvic structure may modify the excitability of nearby sensory afferents and immune cells, while shared pelvic neural pathways may further amplify this effect through cross-organ sensitization (19, 21). Thus, cervical inflammatory input could theoretically influence bladder sensation and urinary symptoms through a combination of local inflammatory signaling and neural cross-sensitization, without requiring primary bladder pathology.

### 5.3 Extra-pelvic pain symptoms

Glossodynia, headache, migraine-like symptoms, neck pain, shoulder pain, back pain, and diffuse musculoskeletal pain are less readily explained by direct pelvic cross-sensitization (38). For these distant symptoms, the proposed mechanism involves sustained peripheral inflammatory and afferent input contributing to central sensitization and altered central pain processing. Persistent nociceptive and immune signaling may increase the responsiveness of spinal and supraspinal networks, allowing pain amplification to occur beyond the anatomical territory of the initiating peripheral source (7–9, 29, 35, 40, 41). In the case of glossodynia, neuropathic and centrally mediated mechanisms have also been proposed (15, 16, 25).

### 5.4 Gastrointestinal symptoms

Gastroesophageal reflux, bloating, nausea, appetite change, abdominal discomfort, and altered bowel function may involve autonomic and brain–gut mechanisms (30). The NTS receives vagal visceral afferent input and communicates with autonomic brainstem nuclei, including the dorsal motor nucleus of the vagus, thereby participating in gastrointestinal regulation. Persistent inflammatory or afferent signaling entering central autonomic networks could theoretically modify visceral perception and autonomic output, contributing to gastrointestinal symptom amplification (1, 22, 23).

For lower gastrointestinal symptoms, particularly rectal discomfort, altered bowel sensation, or constipation-like symptoms, an additional pelvic cross-organ mechanism may be relevant. The colon, rectum, urinary bladder, and reproductive organs have overlapping pelvic sensory pathways, allowing persistent inflammatory or nociceptive activity in one pelvic structure to influence sensory processing in another (19, 21). Local paracrine and neuroimmune signaling may further modulate nearby afferent pathways. Therefore, lower bowel symptoms may theoretically reflect a combination of pelvic cross-organ sensitization, local inflammatory signaling, and centrally mediated autonomic regulation.

### 5.5 Autonomic and systemic symptoms

Palpitations, dizziness, insomnia, anxiety-like internal restlessness, fatigue, sweating, temperature sensitivity, and nonspecific malaise may reflect altered autonomic and interoceptive processing rather than direct effects on individual peripheral organs. The NTS is functionally connected with the hypothalamus, parabrachial nucleus, amygdala, insula, and other components of the central autonomic network. Through these connections, persistent visceral and inflammatory input may influence autonomic balance, arousal, sleep, stress responses, and the perception of internal bodily states (1, 11, 12, 24).

This symptom spectrum should not be interpreted as evidence that cervical inflammation causes all of these conditions. Rather, we propose that, in susceptible individuals, persistent cervical inflammatory input may act as one peripheral contributor to a broader amplification process. Pelvic symptoms may preferentially involve local afferent sensitization and pelvic organ cross-sensitization, whereas distant pain symptoms may depend more heavily on central sensitization and altered supraspinal pain processing. Gastrointestinal and autonomic manifestations may additionally involve NTS-centered autonomic and interoceptive networks. Neuroimmune signaling may interact with each of these pathways by maintaining peripheral and central neural excitability (31, 33). Thus, the proposed model is not a single linear cervix-to-symptom pathway, but an interacting network of pelvic afferent, neuroimmune, autonomic, and central sensitization mechanisms. These cervix-specific links remain hypothetical and require prospective mechanistic validation.

## 6 Proposed mechanism

The proposed cervix–vagus–NTS neuroimmune model is illustrated in Figure 1. The model depicts a hypothesis-generating sequence in which chronic cervical inflammation produces persistent local inflammatory signaling, which may activate pelvic and vagal afferent pathways. These signals may then be integrated within the nucleus tractus solitarius (NTS) and related central autonomic networks, potentially contributing to central sensitization, pain amplification, autonomic dysregulation, and systemic symptom expression.

**Figure 1:**
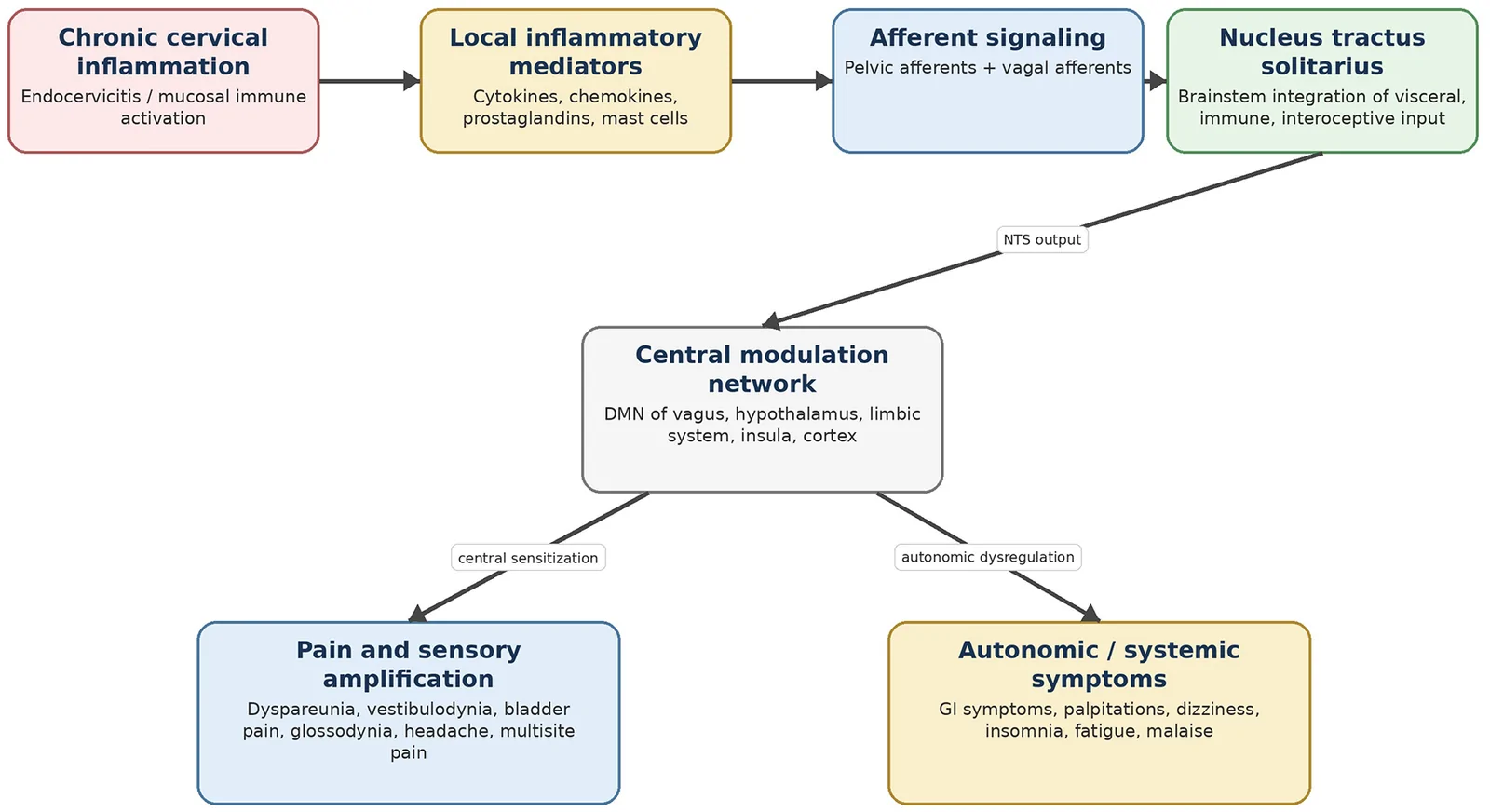
Proposed cervix–vagus–NTS neuroimmune axis. Chronic cervical inflammation may generate persistent local immune activation and inflammatory mediator release. These signals may activate pelvic and vagal afferent pathways projecting to the nucleus tractus solitarius. The NTS integrates visceral, immune, and interoceptive information and communicates with autonomic, hypothalamic, limbic, and cortical networks. Through these pathways, chronic cervical inflammatory input may contribute to pelvic pain, extra-pelvic pain, glossodynia, bladder symptoms, gastrointestinal symptoms, palpitations, dizziness, insomnia, and other systemic neuroimmune manifestations. This model is hypothesis-generating and requires prospective validation.

### 6.1 Level 1: peripheral inflammatory source

Chronic endocervical or cervical inflammation produces local inflammatory mediators, including cytokines, prostaglandins, chemokines, and other immune signals. These mediators may activate local immune cells, nociceptors, and autonomic afferent fibers.

### 6.2 Level 2: afferent neural transmission

Persistent inflammatory signals from the cervix or lower genital tract may be transmitted through pelvic afferents and vagal afferents. Experimental neuroanatomical and electrophysiological studies in rodents support vagal participation in sensory communication between the uterus/cervix and the brainstem (4, 5, 10, 34).

### 6.3 Level 3: brainstem integration

The NTS receives and integrates visceral afferent information. Chronic afferent input from pelvic inflammatory sources may alter NTS activity and influence nearby autonomic nuclei, including the dorsal motor nucleus of the vagus.

### 6.4 Level 4: neuroimmune and autonomic amplification

Through NTS connections with the hypothalamus, amygdala, parabrachial nucleus, insula, and cortical networks, cervical inflammatory input may influence stress responses, pain modulation, arousal, autonomic balance, gastrointestinal function, and immune regulation.

### 6.5 Level 5: clinical symptom expression

The patient may experience pain or symptoms at sites distant from the cervix, including the vulva, bladder, tongue, head, gastrointestinal tract, and musculoskeletal system. This may produce the clinical impression that pain location does not necessarily indicate pathological origin.

As summarized in Figure 1, the proposed model links a focal cervical inflammatory source to distant clinical manifestations through interacting afferent, brainstem, neuroimmune, and autonomic pathways.

## 7 Testable predictions

A strength of this hypothesis is that it generates testable predictions, as summarized in Table 2. Each prediction can be linked to a corresponding testing strategy and biological rationale. These predictions are intended to distinguish association from mechanism and to identify which components of the proposed cervix–vagus–NTS framework are supported or refuted by prospective data. If chronic cervical inflammation contributes to systemic neuroimmune symptoms through the vagus–NTS axis, then future studies should observe one or more of the following:

**Table 2:** Testable predictions of the cervix–vagus–NTS hypothesis.

Prediction	Possible measurement
Cervical inflammation is associated with multisite pain	Gynecologic examination, pain mapping
Treatment of cervical inflammation improves pelvic and extra-pelvic symptoms	Numeric Rating Scale, Marinoff Dyspareunia Scale, glossodynia score
Improvement correlates with autonomic change	Heart rate variability, resting heart rate, autonomic symptom scale
Inflammation correlates with symptom burden	Cervical cytokines, systemic cytokines
Central sensitization modifies treatment response	Central Sensitization Inventory
Vagal modulation influences symptom burden	Heart rate variability change, vagal tone indices

**Prediction 1. Chronic cervical inflammation will be associated with a higher burden of multisystem symptoms**.

Women with objectively defined chronic cervical inflammation are expected to show a higher prevalence or severity of dyspareunia, vulvar pain, bladder symptoms, glossodynia, headache, dizziness, gastrointestinal complaints, sleep disturbance, and multisite pain than appropriately matched women without cervical inflammation. **Testing strategy:** Prospective comparison of women with and without chronic cervical inflammation using standardized symptom inventories and predefined diagnostic criteria. **Rationale:** If a persistent cervical inflammatory source contributes to broader neuroimmune amplification, symptom burden should be greater in women with demonstrable cervical inflammation.

**Prediction 2. Successful treatment of cervical inflammation will be associated with improvement in both pelvic and selected extra-pelvic symptoms**.

Improvement would be expected not only in local pelvic pain but also, in a susceptible subset, in distant symptoms such as glossodynia, headache, dizziness, gastrointestinal symptoms, palpitations, or insomnia. **Testing strategy:** Longitudinal assessment before and after standardized cervical treatment using symptom-specific scales and predefined responder thresholds. **Rationale:** If persistent cervical inflammatory signaling contributes to symptom amplification, reduction of the peripheral inflammatory source should be followed by improvement in at least some downstream symptom domains.

**Prediction 3. Changes in symptom severity will correlate with changes in autonomic function**.

Patients with greater clinical improvement may show corresponding improvement in heart rate variability and other autonomic indices. **Testing strategy:** Measure HRV parameters such as SDNN, RMSSD, and high-frequency power, together with resting heart rate and validated autonomic symptom scales, before and after treatment. **Rationale:** The proposed model implicates NTS-centered autonomic integration; therefore, improvement in symptoms should, if the pathway is relevant, be accompanied by measurable autonomic changes.

**Prediction 4. The severity of cervical inflammation will correlate with local and systemic inflammatory biomarkers**.

Patients with more pronounced cervical inflammatory findings may demonstrate higher levels of inflammatory mediators in cervical secretions and, in some cases, systemic circulation. **Testing strategy:** Quantify cervical and plasma inflammatory markers, including IL-1β, IL-6, TNF-α, and other predefined cytokines, and correlate these levels with clinical inflammation scores and symptom burden. **Rationale:** A neuroimmune model requires evidence that the presumed peripheral inflammatory source is biologically active and that greater inflammatory activity is associated with greater symptom burden.

**Prediction 5. Central sensitization will be greater in patients with broader symptom distribution and may decrease after successful treatment**.

Patients with multisite pain or distant symptoms are expected to demonstrate greater evidence of central sensitization than patients with localized pelvic symptoms only. **Testing strategy:** Use the Central Sensitization Inventory, quantitative sensory testing, pressure-pain thresholds, or other validated measures before and after treatment. **Rationale:** If persistent peripheral input contributes to symptom spread, patients with more widespread symptoms should show stronger evidence of altered central pain processing.

**Prediction 6. Patients with stronger baseline central sensitization may require longer treatment duration or may show incomplete response**.

Patients with greater baseline central sensitization may have a more persistent central amplification state, which could limit or delay symptom improvement after treatment of the peripheral cervical inflammatory source.

**Testing strategy:** Baseline central sensitization should be assessed using the Central Sensitization Inventory and/or quantitative sensory testing. These baseline measures can then be prospectively correlated with treatment duration, magnitude of symptom improvement, and predefined responder status. **Rationale:** If persistent peripheral inflammatory input has already produced a more established central sensitized state, treatment directed at the peripheral cervical inflammatory source may not immediately reverse altered central pain processing. Patients with greater baseline central sensitization may therefore require a longer period of treatment or may show a less complete clinical response.

**Prediction 7. Baseline sensitization and autonomic dysfunction may predict treatment response**.

Patients with greater baseline central sensitization or more marked autonomic dysfunction may require longer treatment or show less complete symptom improvement. **Testing strategy:** Correlate baseline CSI, quantitative sensory testing, HRV, and autonomic symptom scores with treatment duration and predefined responder status. **Rationale:** If the disease process has progressed from a predominantly peripheral inflammatory state to a more established central amplification state, removal of the peripheral trigger alone may produce a slower or incomplete response.

These predictions provide a framework for prospectively testing the proposed cervix–vagus–NTS hypothesis. Future studies should be designed so that failure to observe these predicted relationships would challenge or weaken the proposed model rather than merely generate confirmatory observations.

## 8 Suggested future study design

A prospective controlled study should be designed to test the proposed cervix–vagus–NTS hypothesis while minimizing selection bias, observer bias, regression to the mean, and nonspecific treatment effects. The study should include clearly defined eligibility criteria, an appropriate control group, blinded outcome assessment, standardized treatment protocols, predefined primary and secondary outcomes, and longitudinal follow-up.

### 8.1 Study population

Eligible participants would include women with chronic pelvic pain, dyspareunia, vulvodynia or vestibulodynia, bladder symptoms, glossodynia, or other multisystem complaints in whom chronic cervical inflammation is identified using predefined diagnostic criteria.

Exclusion criteria should include conditions that may independently account for the presenting symptoms, such as active pelvic infection, malignancy, major structural gynecologic disease, severe pelvic floor dysfunction, untreated neurological disease, systemic autoimmune disease, or other clearly established causes of chronic pain.

### 8.2 Comparison groups

At minimum, the study should include a control group of women with comparable chronic pain or multisystem symptoms but without objective evidence of chronic cervical inflammation. A second healthy control group could be included for comparison of inflammatory, autonomic, and sensory biomarkers.

Where ethically and practically appropriate, randomized comparison of active cervical treatment with sham or delayed-treatment control could be considered to distinguish specific treatment effects from placebo response, spontaneous fluctuation, and regression to the mean.

### 8.3 Diagnostic assessment and blinding

Cervical inflammation should be defined prospectively using standardized gynecologic examination and predefined objective criteria, potentially including cervical appearance, cytology, microbiological testing when indicated, and inflammatory biomarkers from cervical secretions.

Whenever feasible, investigators assessing symptom outcomes, autonomic measurements, quantitative sensory testing, and laboratory biomarkers should be blinded to treatment allocation and cervical inflammatory status.

### 8.4 Intervention

Participants undergoing active treatment should receive a standardized cervical treatment protocol with prespecified indications, treatment intervals, and criteria for treatment completion. Concomitant analgesic, anti-inflammatory, or other symptom-directed therapies should be documented and controlled as much as possible.

### 8.5 Primary outcomes

Primary outcomes should be defined before study initiation and may include:

- Change in dyspareunia Numeric Rating Scale;
- Change in Marinoff Dyspareunia Scale;
- Change in glossodynia severity where applicable;
- Change in a predefined multisystem symptom burden score;
- Proportion of participants achieving a predefined clinically meaningful response, such as at least 30% or 50% reduction in symptom severity.

### 8.6 Secondary and mechanistic outcomes

Secondary outcomes may include:

- Heart rate variability, including SDNN, RMSSD, and high-frequency power;
- Resting heart rate and validated autonomic symptom scales;
- Cervical and circulating inflammatory markers, including IL-1β, IL-6, and TNF-α;
- Central Sensitization Inventory scores;
- Quantitative sensory testing;
- Sleep quality and quality-of-life measures;
- Selected small-fiber or autonomic function tests in predefined subgroups.

### 8.7 Follow-up and temporal relationships

Assessments should be performed at baseline, during treatment, immediately after treatment completion, and at prespecified follow-up intervals. Repeated measurements would allow investigators to determine whether changes in cervical inflammation precede, parallel, or follow changes in autonomic function, inflammatory biomarkers, central sensitization, and symptom burden.

### 8.8 Statistical considerations

The primary analysis should compare changes in predefined outcomes between women with and without cervical inflammation and, where applicable, between active-treatment and control groups. Associations between changes in cervical inflammatory markers, autonomic indices, central sensitization measures, and symptom outcomes should be assessed prospectively. Sample-size calculations should be based on the predefined primary outcome.

A prospective controlled study should be designed to test the proposed cervix–vagus–NTS hypothesis while minimizing selection bias, observer bias, regression to the mean, and nonspecific treatment effects. The study should include clearly defined eligibility criteria, an appropriate control group, blinded outcome assessment, standardized treatment protocols, predefined primary and secondary outcomes, and longitudinal follow-up. The key methodological elements of the proposed prospective study design, including comparison groups, blinding, outcome measures, and follow-up strategy, are summarized in Table 3.

**Table 3:** Proposed prospective study design for testing the cervix–vagus–NTS hypothesis.

Study element	Proposed approach	Purpose
Population	Women with chronic cervical inflammation and pelvic or multisystem symptoms	Define target phenotype
Symptomatic control	Similar symptoms without cervical inflammation	Test specificity of cervical inflammation
Healthy control	Optional	Establish biomarker reference values
Intervention	Standardized cervical treatment	Test temporal treatment association
Comparator	Sham or delayed treatment where ethically feasible	Control placebo/regression effects
Blinding	Blinded outcome assessors and laboratory personnel	Reduce observer bias
Primary outcomes	NRS, Marinoff score, multisystem symptom burden, responder rate	Define clinical efficacy signal
Autonomic outcomes	HRV, resting heart rate, autonomic scales	Test autonomic component
Inflammatory outcomes	Cervical and plasma cytokines	Test neuroimmune component
Sensory outcomes	CSI, quantitative sensory testing	Test central sensitization
Follow–up	Baseline, during treatment, post-treatment, long-term	Establish temporal sequence

## 9 Limitations

This hypothesis has several important limitations.

First, the clinical observations described here are preliminary and were presented as conference observations. They do not establish causality.

Second, the relationship between cervical inflammation and systemic symptoms may be confounded by psychological stress, hormonal status, menopause, oral mucosal disease, neuropathy, medication use, infection, autoimmune disease, pelvic floor dysfunction, and other pain syndromes.

Third, the vagus–NTS pathway is biologically plausible, but direct evidence in humans linking chronic cervical inflammation to NTS activation and systemic symptoms remains lacking.

Fourth, improvement after gynecologic treatment does not prove that cervical inflammation was the sole cause of systemic symptoms. Placebo effects, natural fluctuation, nonspecific anti-inflammatory effects, and concurrent behavioral changes must be considered.

Fifth, chronic pain syndromes are heterogeneous. It is unlikely that a single mechanism explains all cases of dyspareunia, vestibulodynia, glossodynia, or multisystem symptoms.

Therefore, this model should be interpreted as a hypothesis-generating framework requiring systematic prospective validation.

## 10 Clinical implications

If validated, this hypothesis may have several clinical implications.

First, clinicians evaluating chronic female pain syndromes may need to consider hidden inflammatory sources outside the symptomatic region.

Second, women with intractable glossodynia, vestibulodynia, bladder symptoms, dyspareunia, or unexplained systemic symptoms may benefit from careful gynecologic evaluation, particularly when conventional local treatment fails.

Third, cervical inflammation should not be viewed only as a local gynecologic condition. In selected patients, it may participate in broader neuroimmune and autonomic dysregulation.

Fourth, the model may encourage interdisciplinary collaboration among gynecologists, pain specialists, neurologists, dentists, oral medicine specialists, urologists, gastroenterologists, and autonomic nervous system researchers.

Finally, this hypothesis may help reframe medically unexplained symptoms in women as potentially arising from measurable neuroimmune and autonomic mechanisms rather than being dismissed as purely psychogenic.

## 11 Conclusion

Pain location may not necessarily indicate pathological origin. Based on two preliminary clinical observations involving dyspareunia and intractable glossodynia, we propose that chronic cervical inflammation may act as a hidden peripheral inflammatory source capable of modulating systemic neuroimmune symptoms through vagal afferent signaling and NTS-mediated autonomic integration.

This hypothesis is not intended as a definitive causal claim. Rather, it provides a clinically motivated and biologically plausible framework for future research. Prospective studies incorporating cervical inflammatory assessment, autonomic measurements, heart rate variability, cytokine profiling, central sensitization indices, and standardized symptom outcomes are needed to determine whether chronic cervical inflammation contributes to systemic symptoms through the vagus–NTS axis.

## Data Availability

The raw data supporting the conclusions of this article will be made available by the authors, without undue reservation.
